# Cancer-associated myositis: a single-centre 20-year experience

**DOI:** 10.1007/s10067-026-08332-3

**Published:** 2026-08-11

**Authors:** Vasiliki Syrmou, Theodora Simopoulou, Eleni Patrikiou, Christos Liaskos, Ioannis Alexiou, Lazaros I. Sakkas, Christina G. Katsiari, Dimitrios P. Bogdanos

**Affiliations:** Department of Rheumatology and Clinical Immunology, Faculty of Medicine, School of Health Sciences, University of Thessaly, University General Hospital of Larissa, Viopolis, Mezourlo Hill, Larissa, 41500 Greece

**Keywords:** Dysphagia, Malignancy, Myositis, Paraneoplastic phenomenon, Rash

## Abstract

**Background:**

Malignancy identified around the onset of idiopathic inflammatory myopathy (IIM) is referred to as cancer-associated myositis (CAM), but population-specific data remain relatively limited.

**Aim:**

To describe the prevalence, clinical features, and risk factors for CAM over a 20-year period in a single tertiary centre in Greece.

**Methods:**

A retrospective analysis was conducted on 113 IIM cases treated between 2001 and 2024 in our department, which is a referral centre for rheumatic disease in Central Greece. Medical records were evaluated for demographics, clinical characteristics, autoantibody profile, and comorbidities. CAM was defined as cases of myositis complicated by the onset or recurrence of cancer within 3 years before or after the diagnosis of myositis.

**Results:**

CAM was identified in 25 (22.1%) patients. Older age and dermatomyositis subtype were associated with CAM. Anti-TIF1γ autoantibody confers significantly higher risk (OR 6.9, *p* < 0.001), while anti-Jo1 lowers risk (OR 0.13, *p* = 0.022). Gottron’s papules and Holster sign were significantly associated with CAM. Raynaud’s phenomenon, interstitial lung disease and the non-interstitial pneumonia pattern appeared protective. Hypothyroidism was less common, while myocardial infarction was more frequent in CAM. Breast, ovarian, and lung cancers were the most common malignancies identified. In most cases, malignancy was asymptomatic (85%) and was determined upon screening. Survival was significantly lower in CAM *p* < 0.001 with the survival rate dropping down to 45% at 31 months vs 95.9% in non-CAM. Median survival was 21 months in CAM cases (1–79 months).

**Conclusion:**

CAM accounts for a substantial proportion of IIM cases. Anti-TIF1γ positivity, older age, absence of Raynaud’s phenomenon, and absence of ILD may serve as clinical flags in our cohort. Comprehensive cancer screening, including GI endoscopy, remains vital in the initial assessment and follow-up, primarily upon relapses and lack of remission.

**Table:** 

**Key Points** • *Population-based studies on cancer-associated myopathy have established a clear, strong link between idiopathic inflammatory myopathies (IIM) and malignancy, often termed cancer-associated myositis (CAM)*. • *This study examines CAM prevalence, features, and risk factors over 20 years at a Greek tertiary center*. • *CAM was present in 22% of IIM patients, mostly asymptomatic (85%), identified through screening; breast, ovarian, and lung cancers were the common malignancies*. • *Akin to previous studies, anti-TIF1γ autoantibody confers a significantly higher risk, while anti-Jo1 lowers risk*.

## Introduction

Idiopathic inflammatory myositides (IIM) are chronic autoimmune conditions affecting muscles and various organ systems, including skin, joints, lungs, heart, and the alimentary tract [1–3]. The IIM family includes dermatomyositis (DM), polymyositis (PM), antisynthetase syndrome, immune mediated necrotizing myositis (IMNM), and inclusion bodies myositis (IBM) [4, 5]. Solid epidemiological data have suggested a link between IIM and cancer and a significant increase in mortality due to malignancies, with up to a tenfold rise in risk within the first-year post-diagnosis [6–8]. IIM patients face a cancer risk of up to 25–32%, particularly within 3 years of diagnosis [9–11]. This remains significantly elevated over the subsequent decade [12]. Conversely, longitudinal data suggest a temporal relationship between certain malignancies and IIM, with colorectal, lung, and ovarian cancers often preceding IIM onset [13]. This indicates a nuanced bidirectional relationship that warrants deeper investigation into the interconnected oncogenic and autoimmune pathophysiological pathways.

Cancer-associated myositis (CAM) is linked to various malignancies, including lung, ovarian, breast, colorectal, and nasopharyngeal cancers, as well as lymphoma [12]. Notably, nearly 80% of CAM cases present with advanced-stage malignancies leading to a poor prognosis and a low remission rate [14].

Given these considerations, understanding the interplay between myositis and malignancy and identifying specific characteristics and factors associated with increased cancer risk are of paramount importance to facilitate earlier diagnosis and improve outcomes in patients with newly diagnosed IIM. However, the heterogeneous nature of IIM further complicates the identification and validation of reliable risk factors. To address this, the International Myositis Assessment and Clinical Studies Group (IMACS), the largest global multidisciplinary consortium dedicated to IIM research, initiated a project to develop consensus-based cancer screening recommendations for IIM [15, 16]. The resulting meta-analysis identified several high-risk features for underlying malignancy, including dermatomyositis (DM), positivity for anti-transcriptional intermediary factor 1γ (anti-TIF1γ) antibodies [17], and anti-nuclear matrix protein 2 (anti-NXP2) antibodies [18], age > 40 years at IIM onset, refractory disease, dysphagia, and the presence of cutaneous necrosis or ulcerations [15, 16].

This study aims to provide a comprehensive analysis of our two-decade experience with IIM and CAM at a designated University Hospital Referral Centre for rheumatic diseases including IIM. Furthermore, we seek to elucidate the associated risk factors that may correlate with the presence of underlying malignancies within this patient population.

## Material and methods

Hospital medical records for all IIM cases managed in the Department of Rheumatology and Clinical Immunology, the only referral centre for rheumatic conditions in Central Greece (estimated inhabitants 1.2 million) between 2001 and December 2024 were retrospectively reviewed for evidence of malignancy. This study comprised only IIM patients classified based on the 2017 EULAR/ACR classification criteria [19]. Data were collected on patient gender, myositis subtype (DM, PM, antisynthetase syndrome, overlap myositis, IMNM), age at myositis diagnosis, age at malignancy diagnosis, and involvement of muscles, skin, lungs, heart, and gastrointestinal system, as well as comorbidities.

The CDASI score was calculated for both activity, and damage and typical DM skin signs were recorded based on the assessment of medical photography. Muscle involvement was recorded as positive when muscle biopsy, MRI, or electromyography (EMG) provided evidence of muscle inflammation. The MMT-8 score was calculated, and the creatine phosphokinase (CPK) level at diagnosis (mg/dL) was noted.

Lung involvement was considered positive if imaging of the thorax (high-resolution computed tomography) showed evidence of an inflammatory or fibrotic process. The percentage of predicted forced vital capacity (FVC) adjusted for BMI was recorded, along with the imaging pattern, specifically noting the presence of non-specific interstitial pneumonia (NSIP), usual interstitial pneumonia (UIP), honeycombing, or emphysema.

For gastrointestinal involvement, medical records were screened for evidence of swallowing difficulties, and and/or hoarseness were logged. Additionally, data on pulmonary hypertension and myocardial involvement were recorded.

The autoantibody profile was assessed [20], including indirect immunofluorescence for ANA, ELISA for anti-ENA, and myositis-specific (MSA) and myositis-associated (MAA) autoantibodies by line blot analysis (Euroimmun, myositis profile) for a range of autoantibodies: anti-Mi2a, anti-Mi2b, anti-TIF-1γ, anti-MDA5, anti-NXP2, anti-SAE, anti-Ku, anti-PM-Scl100, anti-PM-Scl75, anti-Jo1, anti-SRP, anti-PL7, anti-PL12, anti-EJ, anti-OJ, anti-Ro52, anti-CN1a, anti-HA, anti-Ks, anti-Zo, and anti-FARS B. Moreover, data regarding comorbidities including hypertension (HTN), hyperlipidaemia, diabetes, coronary artery disease, stroke and peripheral arterial disease, atrial fibrillation, hypothyroidism, hyperthyroidism, chronic kidney disease (CKD), depression, aortic aneurysm, acute myocardial infarction and other autoimmune diseases were reviewed.

A study protocol, consistent with the Helsinki Declaration, was approved by the Ethics Committee of our Hospital (protocol No 48685/28/11/2024).

### Statistical analysis

Statistical analysis was conducted for demographics, organ involvement, disease subtypes, and autoantibodies. All statistical calculations were performed using SPSS 25 program while graphs were designed using Graph Pad Prism Software. Differences between groups were tested by chi-square test and Fisher’s exact test (2-sided), two tailed *t*-test (for equality of means, equal variances are not assumed) and the non-parametric Mann–Whitney test, as well as Kaplan–Meier survival curves. Fisher’s exact test was used, followed by post hoc analysis for contingency tables. Logistic regression analysis was done for independent risk factors of malignancy. A Venn diagram was created using the InteractiVenn online tool. A *p*-value less than 0.05 was considered statistically significant.

## Results

A total of 113 patients with IIM were included, and 25 patients (22.1%) with malignancy, identified either 3 years before or within 3 years after the myositis onset, were classified as having CAM and included in the statistical analysis. Six additional patients had cancer, but the time interval between malignancy diagnosis and myositis onset exceeded 3 years, ranging from 8 to 19 years, and were excluded from further analysis.

### Age and gender

Patients with malignancy were diagnosed at an older age compared to those without malignancy (mean age 67.92 ± 8.96 vs 57.43 ± 12.68, *p* < 0.001). No gender differences were noted among patients with CAM (Table 1).

**Table 1:** Clinical characteristics and mean laboratory test values of CAM and non-malignancy cases

Clinical characteristics	CAM (*n* = 25)	Non-malignancy (*n* = 82)	*p*-value
Gender (females, %)	14 (56%)	56 (68%)	0.373
Age (mean ± SD)	67.92 ± 8.96	57.43 ± 12.68	< 0.001
Skin Involvement (yes/no)	16/9	46/36	0.639
Type of disease (DM/PM/ARS/OM/IMNM)	16/4/4/0/1	29/6/29/13/5	**0.006**
Lung involvement (yes/no)	5/20	41/41	**0.015**
% pred FVC (mean ± SD)	87.52 ± 13.92	82.99 ± 13.92	0.202
CT thorax pattern (NIL/NSIP/UIP/HC/EMPH)	20/1/3/0/1	41/30/9/1/1	**0.007**
Muscle involvement (yes/no)	24/1	74/8	0.682
CPK (U/L) (mean ± SD)	1,336 ± 1614	2,455 ± 3813	0.950
MMT-8 (mean ± SD)	118.9 ± 25.5	127.35 ± 20.12	0.139
Dysphagia (yes/no)	10/15	20/62	0.205
Voice change (yes/no)	8/17	17/65	0.370
Arthritis/Arthralgias (yes/no)	14/11	54/28	0.085
Raynaud’s Phenomenon	2/23	25/57	**0.033**^**a**^
LDH (mg/dl) mean ± SD	481 ± 261	477 ± 276	0.242
CRP (mg/dl) mean ± SD	1.97 ± 1.78	2.53 ± 3.54	0.292
ESR mean ± SD	47 ± 22.7	45 ± 63	0.81
Ferritin (mg/dl) mean ± SD	403 ± 249	667 ± 992	0.72
WBC count	8501 ± 4126	8867 ± 3768	0.694
Hb (g/l)	12 ± 1.43	12.36 ± 1.58	0.290

^a^, negative association; Statistically significant p-value < 0.05, DM dermatomyositis, PM polymyositis, ARS antisynthetase syndrome, OM overlap myositis, IMNM immune mediated necrotizing myositis, FVC forced vital capacity, CT computer tomography, NIL normal, non NSIP non-specific interstitial pneumonia, UIP usual interstitial pneumonia, HC honeycombing, EMPH emphysema, CPK creatine phosphokinase, MMT-8 Manual Muscle Test-8

### Disease subtypes

**Fig. 1:**
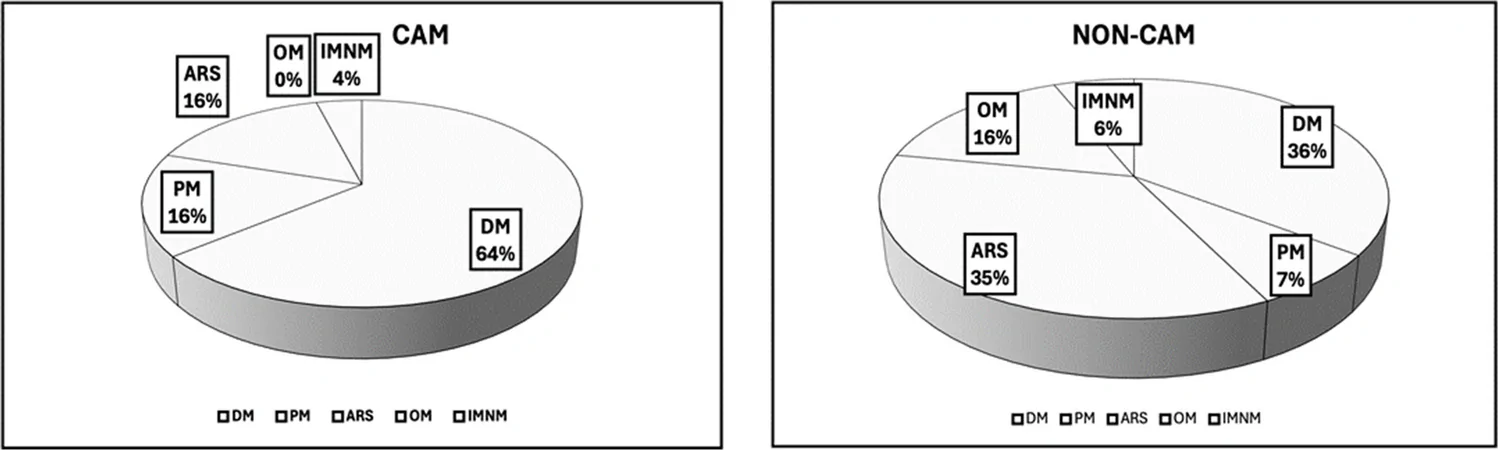
Pie charts showing disease subtype percentages distribution across CAM and non-CAM cases. *DM* dermatomyositis, *PM* polymyositis, *ARS* anti-synthetase syndrome, *OM* overlap myositis, *IMNM* immune mediated necrotizing myositis

CAM was found to be significantly corelated to disease subtype (chi-square for independence, *p* = 0.006). Post hoc analysis with standardized residuals revealed significance level as > + 1.96 and no disease type reached this level. However, maximum trend was seen for DM (+ 1.74) and minimal trend for OM − 1.76 (no OM cases with CAM) (Table 1, Fig. 1). Separate analysis for DM distribution between the CAM and non-CAM showed that DM was unevenly distributed, with a higher percentage in the CAM group (64% vs 35.4%, *p* = 0.021).

### Autoantibodies

**Fig. 2:**
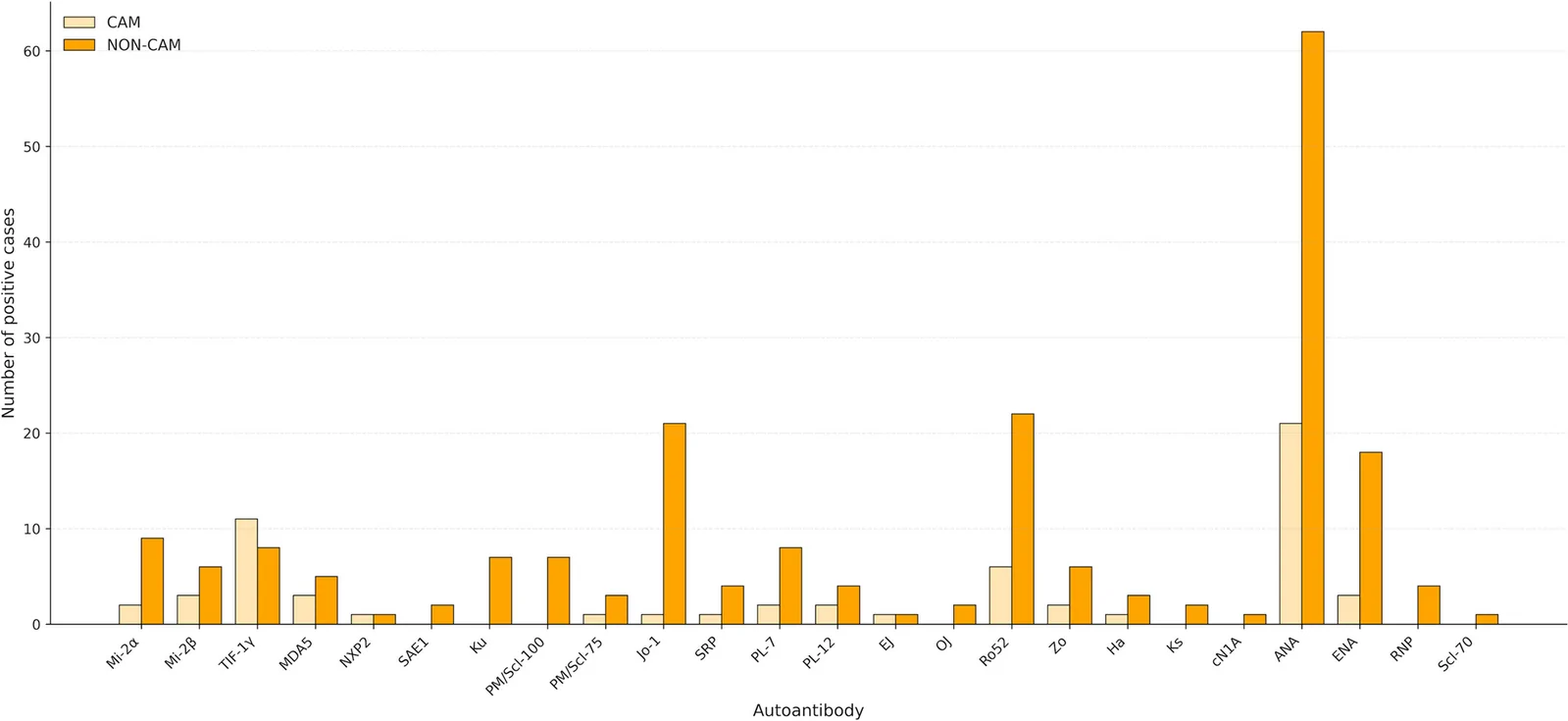
Bar chart revealing the existence of each autoantibody in CAM (yellow) and non -CAM cases (orange bars)

The prevalence of autoantibodies in CAM and non-CAM IIM is shown in Fig. 2. Among MSA and MAA autoantibodies, a statistically significant positive correlation was found between anti-TIF1γ autoantibody and malignancy (OR = 6.90, 95% CI = 2.42–19.68, *p* < 0.001).

Conversely, a negative correlation was found between the classic antisynthetase autoantibody, anti-Jo1, and malignancy (OR = 0.126, 95% CI = 0.016–0.979, *p* = 0.022). No other statistically significant associations were identified (Table 2).

**Table 2:** Myositis related autoantibodies in CAM and non-malignancy cases

Autoantibodies (yes/no)	CAM (*n* = 25)	Non-malignancy (*n* = 82)	*p*-value
anti-Mi-2aanti-Mi-2a	2/23	9/73	1
anti-Mi-2banti-Mi-2b	3/22	6/76	0.433
anti-TIF-1gammaanti-TIF-1gamma	11/14	8/74	** < 0.001**
anti-MDA5anti-MDA5	3/22	5/77	0.386
anti-NXP2anti-NXP2	1/24	1/81	0.414
anti-SAE	0/25	2/80	1
anti-Ku	0/25	7/75	0.196
anti-PM-Scl100	0/25	7/75	0.196
anti-PM-Scl75	1/24	3/79	1
anti-Jo-1	1/24	21/61	**0.022**
anti-SRP	1/24	4/78	1
anti-PL-7	2/23	8/74	1
anti-PL-12	2/23	4/78	0.623
anti- EJ	1/24	1/81	0.414
anti-OJ	0/25	2/80	1
anti-Ro-52	6/19	22/60	0.983
anti-Zo	2/25	6/80	1
anti-Ha	1/25	3/80	1
anti-Ks	0/25	2/80	1
anti-Cn-1A	0/25	1/80	1
anti-FARS B	1/25	3/80	1
ANA	21/4	62/20	0.584
ENA	3/22	18/64	0.391
anti-RNP	0/24	4/73	0.57*
anti-Scl-70	0/23	1/74	1*

Statistically significant p-value <0.05 *test was available in 98 patients. ANA antinuclear antibody, ENA extractable nuclear antigens

### Skin involvement

Skin manifestations were observed in 62 cases (58%) classified as either DM, ARS, or OM. There was no significant corelation between CAM and skin involvement (*p* = 0.639). Total CDASI score in patients with did not differ significantly between the malignancy and non-malignancy subgroups (*t* = 1.53, *p* = 0.145). The mean CDASI total score in CAM group (*n* = 16) was 25.44 ± 16.44 SD vs 18.11 ± 13.02 SD in the non-CAM group (*n* = 46) (Table 2).

However, the classic skin signs Gottron’s papules, heliotrope sign, and Holster sign were found to be significantly and positively associated to CAM (*p* = 0.049, *p* = 0.048, *p* = 0.015, respectively). On the other hand, Raynaud’s phenomenon showed a strong negative association with CAM (*p* = 0.033, 8% in CAM vs 30.5% in non-CAM) (Table 3).

**Table 3:** Typical skin signs in patients with skin involvement with CAM and non-malignancy cases

Main skin signs	CAM (*n* = 16)	Non-malignancy (*n* = 46)	*p*-value
CDASICutaneous Dermatomyositis Disease Area and Severity Index total (mean ± SD)	25.44 ± 16.44	18.11 ± 13.02	0.113
Gottron’s sign (yes/no)	13/3	27/19	0.135
Gottron’s papules	15/1	29/17	**0.024**
V-sign	15/1	33/13	0.09
Heliotrope sign	15/1	32/14	**0.048**
Photosensitivity	13/3	30/16	0.223
Skin ulceration	6/10	13/33	0.585
Shawl sign	7/9	19/27	1
Holster sign	7/9	6/40	**0.025**
Facial erythema	11/6	32/14	1
Alopecia	2/14	10/36	0.71
Scarring	1/15	6/40	1
Depigmentation	2/14	15/31	0.313
Periungual erythema	9/16	31/51	1
Mechanic’s hands	4/21	14/68	0.72
Periorbital oedema	14/11	29/53	0.108
Mucosal lesions	3/22	10/72	1
Sleeve sign (yes/no)	6/19	7/75	0.12

### Lung involvement

A strong negative association was identified between malignancy and interstitial lung disease (ILD) (*p* = 0.15). However, the independent *t*-test for the percentage of predicted % FVC value and malignancy showed no significant correlation (*t* = 1.19, *p* = 0.202).

As for CT thorax patterns of lung involvement, nearly 80% of malignancy cases had a normal CT thorax, while 12% exhibited a UIP pattern, 4% had an NSIP pattern, and 4% had emphysema. In the non-malignancy group, these patterns were observed in 50%, 36.5%, 11%, and 1.2% of cases, respectively. The CT thorax was found to have statistical corelation with malignancy *p* = 0.007 (by the chi-square test for independence). From post hoc analysis, the NSIP pattern was shown to have a rather protective (negative) correlation with malignancy (standardized residual = − 2.33 < 1.96) (Table 1).

### Muscle involvement

No significant differences in muscle involvement were observed between the groups. Comparison of MMT-8 scores between the malignancy and non-malignancy groups showed no significant difference (118.9 ± 25.5 SD vs 127.35 ± 20.12 SD, *p* = 0.139). Similarly, no significant differences were found in CPK levels at myositis diagnosis between CAM and non-CAM groups (Mann–Whitney test, mean CPK 1336 U/L ± 1614 SD in CAM vs mean CPK 2455 U/L ± 3813 SD in non-CAM, *p* = 0.95) (Table 1).

### Difficulty in swallowing and voice changes

In our IIM cohort, 32 patients (28.3%) experienced difficulty in swallowing. Among them, 10 patients (40%) had CAM (*p* = ns) and 25 patients (23.3%) experienced voice changes, with 8 (32%) of those occurring in the CAM group (*p* = ns) (Table 1).

### Heart involvement, arthritis and arthralgias

No significant association of CAM with heart involvement or arthritis/arthralgias was found (Table 1).

### Comorbidities

A comparison between patients with and without malignancy revealed no significant associations, except for hypothyroidism and acute myocardial infarction. Hypothyroidism was less common in patients with malignancy (*p* = 0.007), whereas myocardial infarction was positively associated with malignancy (*p* = 0.031).

### Laboratory test results

Among laboratory features, including ferritin, CRP, LDH, WBC, and Haemoglobin at diagnosis, no statistically significant association with CAM was found (*p* = ns) (Table 1).

### Types of malignancy

**Fig. 3:**
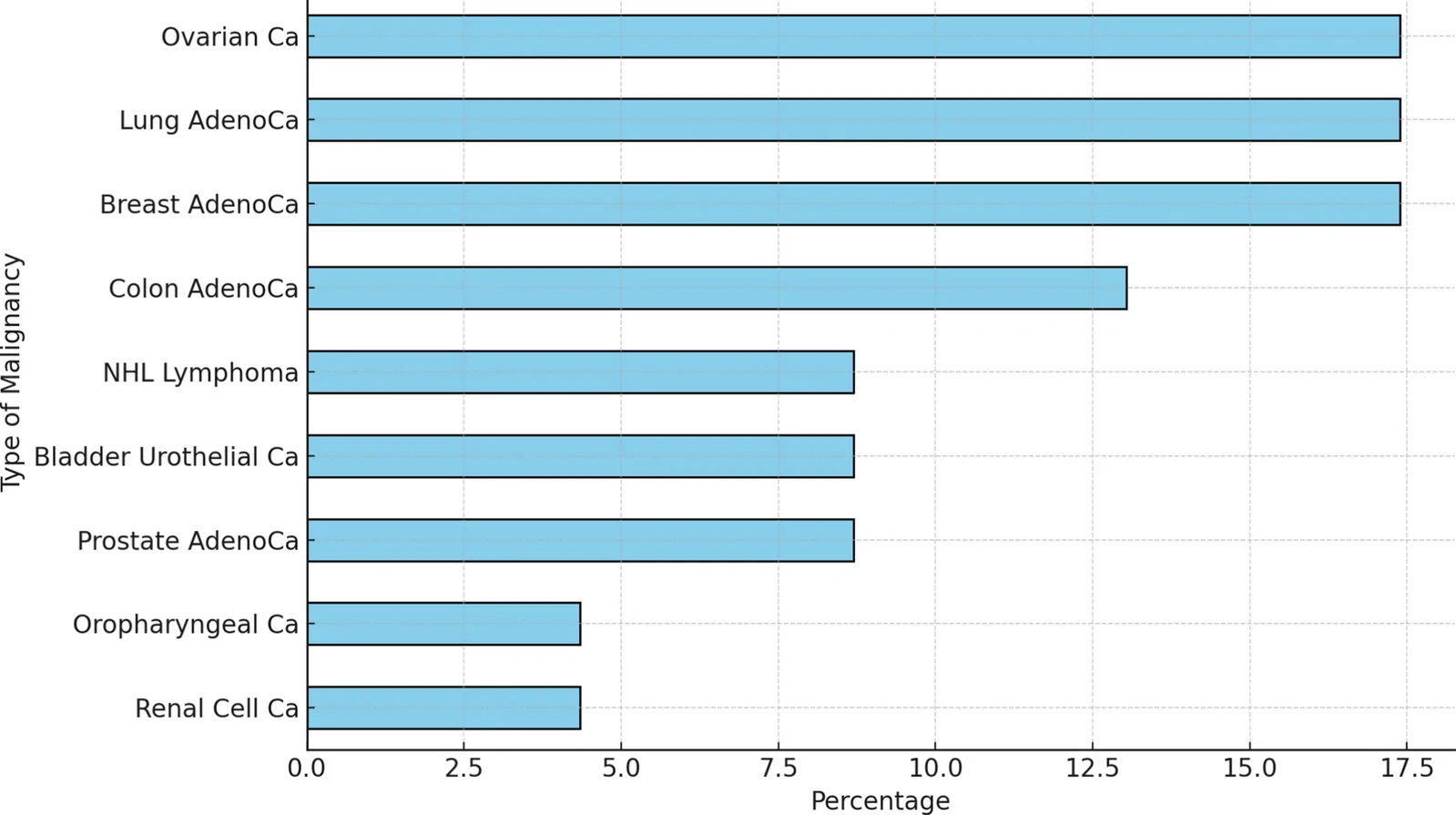
Bar chart revealing the percentages of each malignancy type in our cases with idiopathic inflammatory myopathies. Ca carcinoma, NHL Non Hodgkin lymphomaBar chart revealing the percentages of each malignancy type in our cases with idiopathic inflammatory myopathies. Ca carcinoma, NHL Non Hodgkin lymphoma

A bar chart revealing the percentages of each malignancy type in our IIM cases is shown in Fig. 3.

The most frequently observed cancers were breast cancer (4 cases, 17.4%); ovarian cancer (4 cases, 17.4%); and lung cancer (4 cases, 17.4%), followed by colon cancer (3 cases, 13%); prostate cancer (2 cases, 8.7%); urothelial bladder cancer (2 cases, 8.7%); B-cell non-Hodgkin lymphoma (2 cases, 8.7%); and gastric cancer (2 cases, 8.7%). Additionally, there was one case each of renal cell carcinoma (4.3%) and oropharyngeal cancer (4.3%). Among female patients, the most common malignancies were breast cancer (4 cases) and ovarian cancer (4 cases), followed by non-Hodgkin lymphoma (2 cases), gastric cancer (2 cases), and oropharyngeal cancer (1 case). In male patients, lung cancer was the most frequently observed malignancy (4 cases), followed by colon cancer (2 cases), prostate cancer (2 cases), urothelial bladder cancer (2 cases), and renal cell carcinoma (1 case).

### Timing of myositis and malignancy diagnosis

Among the 25 CAM patients, myositis was diagnosed more than 6 months before malignancy in 10 cases, with an average interval of 20.8 months between diagnoses. In 5 cases, myositis was diagnosed after malignancy, with an average interval of 18.7 months. For the remaining 10 cases, the two diagnoses were considered synchronous, as they occurred within a 3-month interval. No significant associations were identified between malignancy type and the timing of myositis diagnosis. Excluding the 5 metachronous cases, 17 cases (85%) of the rest were asymptomatic for malignancy (apart from the IIM symptoms) and malignancy was diagnosed either upon initial screening (appropriate for age and gender) or on IIM relapse or lack of response to IIM treatment (Table 4).

**Table 4:** Timing, types of CAM, and survival of patients

A IIM type	Type of malignancy	Follow up (survival in months)
DM (2)	Lung (1), bladder (1)	Remission in 2 cases (> 42/ > 6)
PM (1)	NHL	Partial response (> 14)
ASS (2)	Lung (1), breast (1)	Refractory in 1 case (10) Partial response in 1 case (31)
B IIM type	Type of malignancy	Follow up (survival in months)
DM (8)	Ovarian (3), breast (2) Oropharyngeal (1)	Refractory in 7 cases (10/8/1/21/18/3/6) Initial response in 1 case (10)
ASS (1)	Lung	Partial response (> 79)
PM (1)	Prostate	Refractory (2)
C IIM type	Type of malignancy	Follow up (survival in months)
DM (5)	Colon (1), breast (1) Gastric (2), Prostate (1)	Partial Remission in 1 case (> 40) Remission in 1 case (> 48) Refractory in 3 cases (33/ > 34/35)
ASS (3)	Ovarian (1), NHL (1) ONE MISSING	Partial Response in 1 case (46) Initial response followed by refractory relapse in 1 case (14) Refractory in 1 case (14)
PM (1)	Colon (1)	Initial response, relapse at year 1 and partial Remission (59)
IMNM (1)	Bladder (1)	Initial complete response, relapse at year 3 (> 56 months)

(A) CAM metachronous to malignancy. (B) CAM synchronous to malignancy. (C) CAM preceding the diagnosis of malignancy. IIM Idiopathic inflammatory myopathies, *DM* dermatomyositis, *PM* polymyositis, *ASS* antisynthetase syndrome, *IMNM* immune mediated necrotizing myositis, *NHL* non-Hodgkin lymphoma, > : patient is alive

### Treatment

Therapeutic decision was guided by evidence of malignancy upon initial screening. If malignancy was present upon diagnosis minimal immunosuppressive treatment was given except cases with severe manifestations like severe muscle weakness or dysphagia. The mainstay of treatment in CAM is the treatment of underlying malignancy. For the rest of the cases treatment was given according to main manifestation up to malignancy diagnosis. Intravenous immunoglobulin (IVIG) was a helpful tool for patients with CAM and very compromised performance status due to muscle weakness or dysphagia (even in lower doses). Chemotherapy was decided and administered by oncologists. In two cases, immunotherapy with PD-1/PDL-1 inhibitors were initiated after CAM diagnosis. Of those patients, 1 case with breast cancer is free of malignancy (> 34 months), and myositis is in remission, while the other patient had lung cancer and was started on pembrolizumab with rapid deterioration due to malignancy (however, there was significant improvement in DM rash and muscle enzymes).

### Survival

**Fig. 4:**
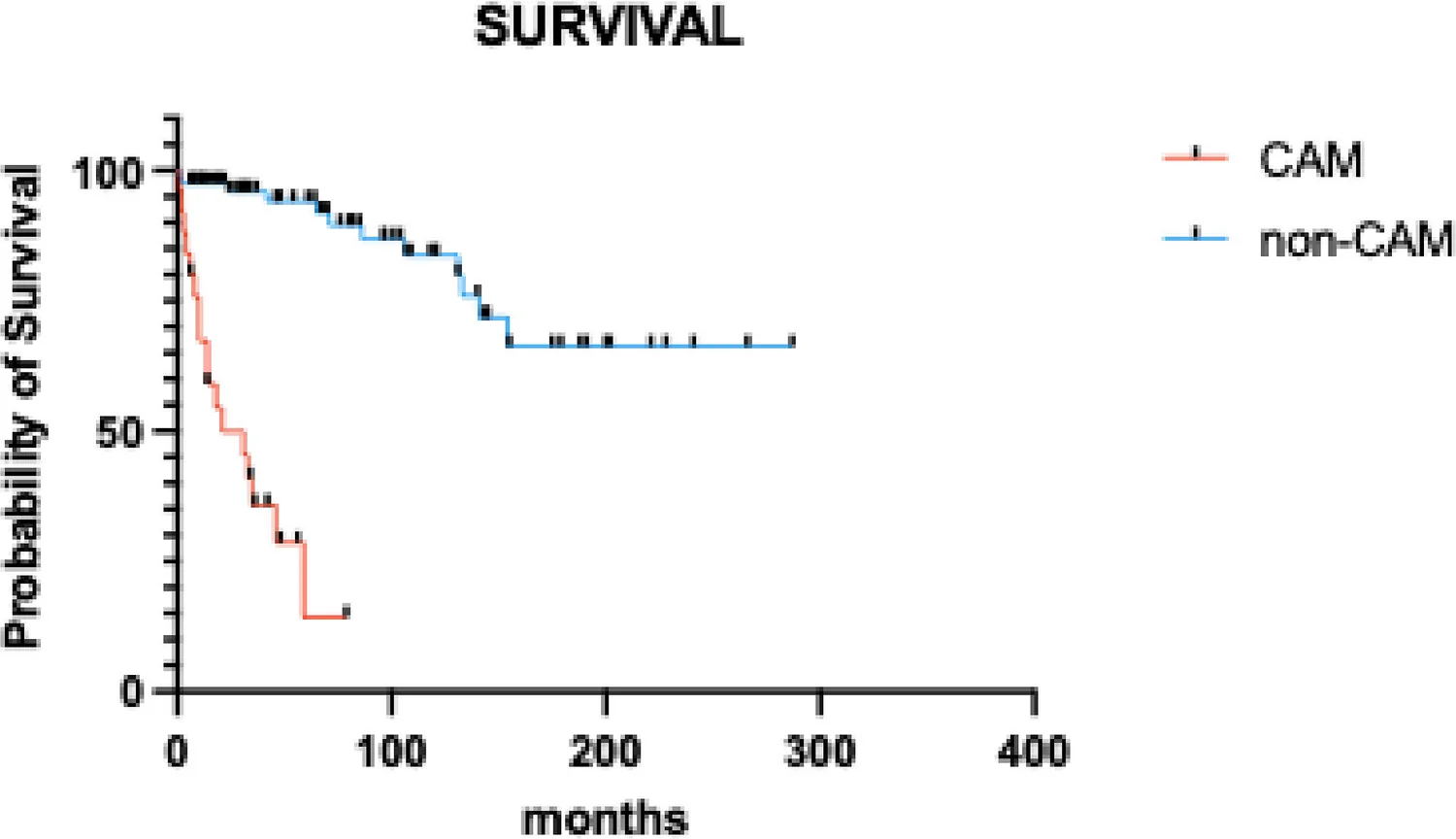
Survival Curves in patients with CAM vs non-CAM. Kaplan Meier survival curves in CAM (red curve) and non-CAM (blue curve) cases. The 2 curves are significantly different *p* < 0.001

In terms of survival, CAM and non-CAM IIM cases differed significantly *p* < 0.001. The 6-month survival rate was 80% for CAM vs 97.53% for non-CAM, the 10-month survival was 67.37% vs 97.53%, and the 31-month survival was 45% vs 95.9%. CAM median survival was 21 months (Fig. 4).

## Discussion

In the present study, we describe our single centre 20-year experience on CAM, in a Department of Rheumatology, a tertiary centre in central Greece. IIMs represent a heterogeneous group of autoimmune disorders with a well-documented association with malignancy [11]. Prior to this work, we have recently reported (in accordance with several published data) that in our series, CAM was three times more frequent in the anti-TIF1γ antibody positive patients with IIM compared to the seronegative group [20]. In our present study, most malignancies were identified around the time of IIM diagnosis. A small number of cases (*n* = 6). The prevalence of CAM was 22.1%, which agrees with previously published data [9, 10]. Our findings further confirmed that CAM patients tend to be older at the time of diagnosis compared to IIM patients, with a mean age of approximately 67 years. Although the overall cohort demonstrated a female preponderance in line with other IIM studies, gender did not appear to correlate with CAM risk within our cohort. This may be due to the relatively small cohort size of our cohort.

In previous studies DM consistently demonstrated the strongest association with malignancy [1, 11, 15, 16, 21–23]. Although, a positive trend was also noted in our cohort; it did not reach significance levels. However, DM cases were disproportionally more in CAM group. It is important to note that patients exhibiting a classic DM rash were classified as anti-synthetase syndrome (ARS) or OM if they tested positive for anti-synthetase antibodies, which may have influenced this result. An additional noteworthy finding was the absence of CAM among patients with OM features, potentially suggesting a protective role for this subtype, but this requires external validation. Furthermore, Raynaud’s phenomenon was found in nearly all OM cases in our cohort. Interestingly, Raynaud’s phenomenon demonstrated a statistically significant negative association with malignancy, raising the possibility of distinct immunopathogenic mechanisms underlying CAM compared to IIM in general [24].

As expected, anti-TIF1γ autoantibody was confirmed to have a strong correlation with CAM, with the risk exceeding 50% in anti-TIF1γ-positive cases [20]. However, the previously reported elevated risk associated with anti-NXP2 positivity [18] was not corroborated in our cohort. Interestingly, the presence of anti-Jo1 autoantibodies shows a negative correlation with CAM, which is consistent with data presented at IMACS 2023, although its presence does not entirely exclude the possibility of CAM.

Gottron’s papules, heliotrope, and Holster sign were also found to be associated with a higher risk of CAM. However, CDASI score did not correlate with CAM risk. This finding aligns with the separate analysis focused on skin ulcerations, which also failed to demonstrate an association with CAM-somewhat contrary to expectations based on IMACS 2023 data [15, 16].

Our findings indicate that lung involvement was associated with a lower risk of CAM. Notably, the presence of a NSIP pattern appeared to confer a protective effect against malignancy, including lung cancer. Muscle involvement was similar across groups, whereas CAM patients had lower CPK. Finally, dysphagia was not significantly different in CAM patients, possibly due to small sample size. Autoimmune hypothyroidism was significantly more common in non-CAM patients, showing a negative association with malignancy. In contrast, CAM patients had a higher prevalence of acute coronary syndromes.

The close temporal relationship between IIM onset and cancer diagnosis suggests a complex pathogenic interplay between these conditions, including genetics, tumour mutations, and autoimmunity [13]. This complexity is further underscored by the diverse and often unpredictable clinical manifestations, the wide spectrum of implicated autoantibodies, and the observation that certain IIM subtypes exhibit a stronger risk for malignancy than others [16–18].

Although not much is known regarding pathogenesis of IIM, it is well understood that for every IIM case, it is of paramount importance to perform an appropriate risk assessment, including demographics, manifestations, serology, and inflammatory markers, as well as an age—and population-appropriate malignancy screen as recommended by IMACS [16] and local protocols.

## Data Availability

Raw data can be provided to third parties upon justified request.

## Abbreviations

IIM: Idiopathic inflammatory myopathies
ILD: Interstitial lung disease
DM: Dermatomyositis
PM: Polymyositis
IBM: Inclusion bodies myositis
IMNM: Immune-mediated necrotizing myopathy
ARS: Anti-synthetase syndrome
OM: Overlap myositis
MSA: Myositis specific autoantibodies
MAA: Myositis associated autoantibodies
CAM: Cancer associated myositis
TIF1: Transcription intermediary factor1
TGFβ: Tumour growth factor β
CDASI: Cutaneous Dermatomyositis Disease Area and Severity Index
MRI: Magnetic reasoning imaging
EMG: Electromyogram
MMT-8: Manual Muscle Test-8
CPK: Creatine phosphokinase
FVC: Forced vital capacity
NSIP: Non-specific interstitial pneumonia
UIP: Usual interstitial pneumonia
HTN: Hypertension
CKD: Chronic kidney disease
SD: Standard deviation
PAD: Peripheral arterial disease
CAD: Coronary artery disease
LDH: Lactate dehydrogenase
WBC: White blood cells
Hb: Haemoglobin
CRP: C-reactive protein
ESR: Erythrocyte sedimentation rate
